# Inhibition of AMPK expression in skeletal muscle by systemic inflammation in COPD rats

**DOI:** 10.1186/s12931-014-0156-4

**Published:** 2014-12-07

**Authors:** Yong Qi, Jun-yi Shang, Li-jun Ma, Bei-bei Sun, Xin-gang Hu, Bao Liu, Guo-jun Zhang

**Affiliations:** Department of Respiratory and Critical Care Medicine, Zhengzhou University People’s Hospital (He’nan Provincial People’s Hospital), 7 Weiwu Road, ZhengZhou, 450003 China; Department of Respiratory and Critical Care Medicine, The First Affiliated Hospital of Zhengzhou University, 1 Jianshe East Road, ZhengZhou, 450003 China

## Abstract

**Background:**

Chronic obstructive pulmonary disease (COPD) is a disease characterized by airflow limitation and inflammation. Meanwhile, COPD also is associated with metabolic disorders, such as skeletal muscle weakness. Strikingly, activation of AMP-activated protein kinase (AMPK) exerts critical roles in energy metabolism. However, it remains unclear whether and how the expression levels of AMPK are affected in the COPD model rats which may lead to the dysfunction of the skeletal muscle in these rats.

**Methods:**

Here we developed a rat model of COPD, and we investigated the morphological changes of peripheral skeletal muscle and measured the levels of tumor necrosis factor -α (TNF-α) and AMPK in skeletal muscle by using approaches that include immunohistochemistry and polymerase chain reaction (PCR).

**Results:**

We found that the expression levels of both AMPK mRNA and protein in skeletal muscles were significantly reduced in the COPD model rats, in comparison to those from the control rats, the COPD model rats that received treatments with AICAR and resveratrol, whereas the expression levels of TNF-α were elevated in COPD rats.

**Conclusion:**

Such findings indicate that AMPK may serve as a target for therapeutic intervention in the treatment of muscle weakness in COPD patients.

## Background

Chronic obstructive pulmonary disease (COPD) is characterized by a kind of airflow limitation that is not fully reversible [1]. The airflow limitation is usually progressive and associated with an abnormal inflammatory response of the lungs [2]. However COPD is also associated with comorbidities such as metabolic diseases, sleep apnea, anemia, weight loss and skeletal muscle weakness. In according to the World Health Statistics report (2013) that these comorbidities are progressively increasing healthcare burden and hospital admission, which lead to a reduced health status and increasing mortality in addition to the dysfunction of lung. It is reported that COPD currently is the fourth leading cause of death in the world, and will rise to the third leading cause of death by 2030 [3]. Furthermore, it has been well recognized that the dysfunction of skeletal muscle is an independent predictive factors leading to death [4]. Skeletal muscle weakness is reflected by the reduced muscle strength and endurance, as well as the increased muscle fatigability [5]. Muscle weakness is mainly observed in the lower limb muscle of patients with COPD [6]. For instance, quadriceps muscle weakness is a common feature in patients at all stages of COPD [7,8]. During the period of acute exacerbations of COPD, the strength of upper limb muscles is also noticed to be reduced [9]. With the development of COPD,the performances of lower extremity muscles include *(i)* muscle fiber type shifting from type I towards type IIx muscle fibers which results in reduced oxidative and fiber atrophy; *(ii)* increasing glycolytic capacity; *(iii)* loss of muscle mass; and *(iv)* decreased capillary density. Collectively, it will be a useful predictor for mortality in patients with COPD [10].

Meanwhile, recent studies suggest that systemic inflammation exerts critical roles in the development of COPD. Clinical studies demonstrate that COPD patients have apparent skeletal muscle dysfunction, in addition to the difficulties in breathing. However, the relationship between energy metabolism and the inflammation implicated in COPD patients remains unclear. In COPD patients,the serum levels of several inflammatory mediators were increased,such as TNF-α [11], interleukin (IL)-1β [12], IL-6, IL-8, IL-18 [13], and acute phase reactants [14]. In the COPD patients who are hospitalized due to acute exacerbations, the serum levels of IL-8 are increased and also negatively associated with the weakness in the quadriceps. It has been indicated that pharmacological activation of AMPK, e.g. by AICAriboside (AICAR), exerts negative influential effects on the inflammatory processes through increasing the release and/or the production of anti-inflammatory cytokines. It has also been shown that activation of AMPK can increase macrophage phagocytosis. Conversely, the obesity and/or LPS treatment-based reduction of the AMPK activities leads to the increase in the pro-inflammatory responses, which may precede insulin resistance. It is also reported that inhibition of inflammation may result in more healthy aging and prolonged life [15]. Meanwhile, AMPK is a key sensor and/or regulator of cellular energy. Once activated, AMPK switches on the catabolic pathways to generate adenosine triphosphate (ATP) whereas AMPK switches off the biosynthetic pathways that consume ATP. Thus, the ATP levels are elevated when the AMPK is activated. Because the cellular energy status is a crucial factor for the cells to exert their diverse functions in all aspects of cell function, it is not surprising that AMPK has umpteen downstream targets whose phosphorylation mediates dramatic changes in cell metabolism, cell growth, and other functions. Pharmacological activation of AMPK by metformin holds a therapeutic potential to reverse metabolic abnormalities such as type-2 diabetes and nonalcoholic fatty liver disease. However, it remains unclear about the functional roles for the AMPK activities in the skeletal muscle dysfunction complicated with COPD. Therefore, to address these issues, we established the COPD model rats. We observed morphological changes of peripheral skeletal muscle as well as the abnormalities of mitochondria in skeletal muscle in these COPD rats. We also measured the expression levels of TNF-α and AMPK in the skeletal muscle in COPD rats, and found that the treatments with resveratrol and/or AICAR prevented the reduction of the AMPK levels in COPD rats in comparison to those in the control rats. Collectively, these results suggest that AMPK may help develop therapeutic strategies in the treatment of muscle weakness in the COPD patients.

## Methods

### Reagents

Hongqi Canal cigarettes (tobacco type of tar: 14 mg, nicotine content: 1.2 mg carbon monoxide fumes: 15 mg) were from Henan tobacco industry limited liability company; LPS and AICAR were purchased from Sigma. Bo compound resveratrol capsules Litas (LTL; Shanghai); TNF-α ELISA kit from PeproTech; AMPKα1 rabbit anti-mouse antibodies and the secondary antibodies were purchased from Santa Cruz. Phospho-AMPKα1 (Ser485) rabbit antibodies were purchased from ABGENT. SirT1 (D1D7) rabbit mAb was purchased from Cell Signaling Technology.

### Animal treatment

Male Wistar rats (200 ± 20 g) were purchased from Henan Experimental Animal Center (Zhengzhou). These animals were housed in a temperature- and humidity-controlled condition and kept on a 12 hr/12 hr light/dark cycle, with free access to regular chow and water. Rats were randomly divided into four groups with fifty rats per group, as described below: control group, COPD + saline group, COPD + AICAR group and COPD + resveratrol group.

### Preparation of COPD model rat

Experimental protocol was approved by the Experimental Animal Care and ethics committee in the First Affiliated Hospital, Henan University of Traditional Chinese Medicine, Zhengzhou, China. The detailed procedures about COPD model rats were described in the previous report [16]. Briefly, the rats were anesthetized using intraperitoneal injection of ketamine (100 mg/kg), and were administered with lipopolysaccharide (LPS) twice through intratracheal instillation on Day 1 (0.2 ml in 1 mg/mL) and Day 15 (0.1 ml in 1 mg/ml) during the procedures. The rats were exposed to tobacco (Hongqi Canal®Filter tip cigarette, tobacco type, tar: 10 mg, nicotine content: 1.0 mg, carbon monoxide: 12 mg, Henan Tobacco Industry, Zhengzhou, China) smoke of 8 cigarettes per treatment, twice a day, during the first two weeks; then fifteen cigarettes per treatment, three times per day, from week 3 through week 12. The rats were placed in a sealed box connected to smoke source to receive two or three times of 30 min exposures per day, at the interval of one or two 3 hr. Control rats were exposed to the air following the similar procedures as described immediately above.

### Administrations

Starting from Day 29, two COPD rats were sacrificed to evaluate if the model was successfully made on the basis of the pathological changes of lung histology and pulmonary functional impairment. The rest rats received control saline and/or chemicals as grouped below: *(i)* Control group (2 ml of 0.9% saline, intragastriceally, once a day for 30 days); *(ii)* COPD model group (2 ml of 0.9% saline, intragastriceally, once a day for 30 days); *(iii)* COPD model group that received AICAR (AICAR; 250 mg/kg, subcutaneously, once a day for 30 days); *(iv)* COPD model group that received resveratrol (Resveratrol, 100 mg/kg, intragastrically, once a day for 30 days).

### Lung and skeletal muscle histology

At the end of the treatments for each rat, lung and skeletal muscle tissues were sampled after euthanasia and cut into 3-millimeter thick slices along the maximum diameter of the right lower lobe and lower extremity and fixed them in 4% paraformaldehyde (PFA) for 72 hours. The samples were embedded with paraffin, and sections of the samples were made in 4 μm thickness. Lung tissue stained with hematoxylin and erosin (H & E) and skeletal muscle stained with MASSON. All images were taken at amplification of 200 under an Olympus PM-10 AD optical microscope and photographic system (Olympus, Tokyo, Japan).

### Skeletal muscle ultrastructure morphology

The skeletal muscle tissues were cut into sections at the thickness of 1-millimeter. The sections were fixed in glutaraldehyde for 2 hours, and then were post-fixed in 1% osmium tetroxide, stained in uranyl acetate, dehydrated in a methanol series and propylene oxide, and embedded in epoxy resin. The sections at 70 nm in thickness were stained with uranyl acetate and lead. The muscle filament arrangement, muscle weakness and mitochondria were analyzed by taking images of the sections using a JEM-1400 transmission electron microscope and photographic system (Hitachi, Japan).

### Estimate the TNF-α in serum and skeletal muscle

The level of TNF-α in serum and skeletal muscle tissue was assayed by Enzyme-linked immunosorbent assay (ELISA) in according to the instruction of the kits (PeproTech, Rocky Hill, United States).

### Real time fluorescence quantitative PCR of AMPKα_1_

For total RNA extraction, rat skeletal muscle (100 mg) were dissolved in Trizol (1 ml) (Life Technologies, NY, USA), and assessed by agarose gel electrophoresis and absorbance measurements at 260 and 280 nanometer (nm) wavelength on SP-1901 ultraviolet light spectrophotometer (Jinpeng Analytic Instrument Company, Shanghai, China). The purification and integrity of the total RNA were assayed. Reverse transcription (RT) was performed by using Supre® III First-Strand Synthesis SuperMix for qRT-PCR (Life Technologies, NY, USA). PCR was performed by using Platinum SYBR® Green® Super Mix-UDG Kit (Life Technologies, NY, USA). The reaction system was prepared following the instructions of the kits. The initial activation was at 95°C for 15 s, 60°C for 30 s, 40 cycle; and 95°C for 15 s, 60°C for 1 min, 95°C for 15 s, 60°C for 30 s (ABI, CA, USA). The relative quantity of mRNA expression was assayed using 2-ΔΔCt formula. The primers of AMPKα_1_ were designed and synthesized by Generay Biotech Co. Ltd. (Shanghai, China). The primer sequences of glyceraldehyde-3-phosphate dehydrogenase (GAPDH) are: forward (5′-3′) gggtcagaaggattcctatg and reverse (5′-3′) ggtctcaaacatgatctggg; and the primer sequences of AMPK are: forward (5′-3′) cattcttggttgccgaaaca and reverse (5′-3′) tgtttggatttctgtgggtt.

### Western blotting of AMPKα_1_, p-AMPKα_1_, SIRT1 analysis

The skeletal muscle tissues were grinded into powder using liquid nitrogen in a mortar. The tissue lysates were collected into EP tube, and were centrifugated with 10000 r/min for 10 min in 4°C. The protein content in the supernatant was measured using BCA (bicinchonininc acid), and the supernatant was stored at -70°C. 100 μg protein from each group of the samples were assayed by SDS-PAGE electrophoresis, and the proteins were transferred to PVDF membrane in a buffer containing 5% skim milk for Tween 20-Tris HCl seal film 4 h at room temperature. The PVDF membrane was incubated with the first antibody, mouse anti- AMPKα1, phospho-AMPKα1(Ser485) antibody and Sirt1(D1D7) rabbit mAb at room temperature for 1 h and then at 4°C incubated for 24 h. After 5 min rinsing with 0.01 mol/L Tween 20-Tris HCl buffer for three times, the PVDF membrane was incubated with horseradish peroxidase-labeled anti-mouse IgG secondary antibody for 1 h at room temperature. The PVDF membrane was rinsed with 0.01 mol/L Tween 20-Tris HCl buffer for 5 min and repeated 3 times. The light emission absorbance of hybridization was detected by using electrogenerated chemilummesence (ECL) and assayed by quantitative image analysis software.

## Results

### Reduction of body weight in COPD model rats

**Figure 1:**
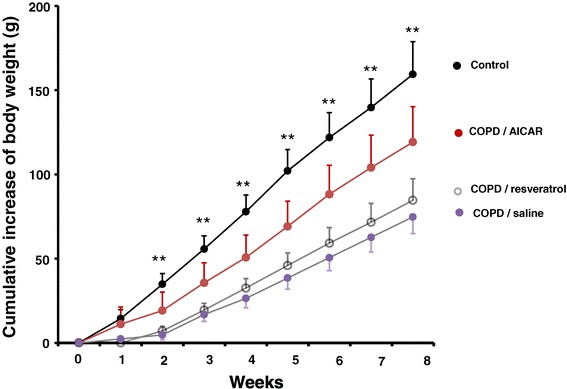
**Decrease of body weight in the COPD model rats.** The body weight of the COPD model rats (filled circles in pink) (n = 13) was significantly reduced, in comparison to the body weight in the control rats (filled circles in black) (n = 12). AICAR treatment (n = 13, filled circles in red) prevented the reduction of body weight in the COPD model rats. **p < 0.01; student *t* test.

In comparison to the control rats, the body weight in COPD model rats was gradually decreased (Figure 1), suggesting that the status of energy metabolism was altered in COPD model rats which was consistent with the decrease in the body weight in COPD patient. Intriguingly, we also noticed that AICAR treatment prevented the reduction in the body weight in the COPD model rats while there were no significant changes of the body weight in the COPD rats treated with resveratrol (Figure 1). Further studies will be needed to investigate the underlying mechanism(s) of the reduction of body weight in the COPD model rats.

### Pathological changes in lung tissue

**Figure 2:**
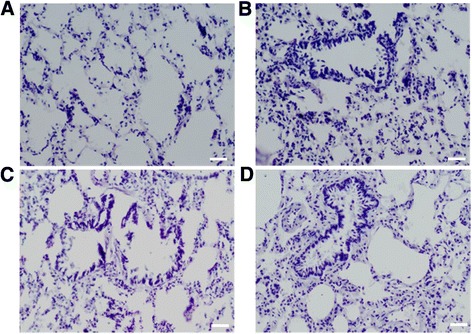
**Pathological changes of the lungs in control rats and COPD model rats that were treated with vehicles, AICAR and/or resveratrol at week 20 (H & E stained). (A)** Control rats; **(B)** COPD rats with vehicle; **(C)** COPD rats with AICAR treatment; and **(D)** COPD rats with resveratrol treatment. Amplification ×200. Scale bar, 20 μm.

The outline of the lung in COPD model rats was enlarged and looked pale on the surface of the lung. The sections of the lung also showed flaky brown smoke spots. As shown in Figure 2, the thickness of bronchiole wall in the COPD model rats was higher than that in the control rats at 20 weeks while the thickness of the alveolar wall was significantly reduced in the COPD model rats that were treated with either AICAR or resveratrol in comparison to the thickness of the wall from the COPD model rats that received the vehicle control saline injections. Furthermore, the structure of the alveolar was destructed as indicated by the extensive alveolar wall thickening, alveolar rupturing and small blood vessel wall thickening under light microscopy (Figure 2B). These abnormalities were ameliorated with the treatment of either AICAR (Figure 2C) or resveratrol (Figure 2D).

### Abnormal morphological changes in skeletal muscle tissue in COPD rats

**Figure 3:**
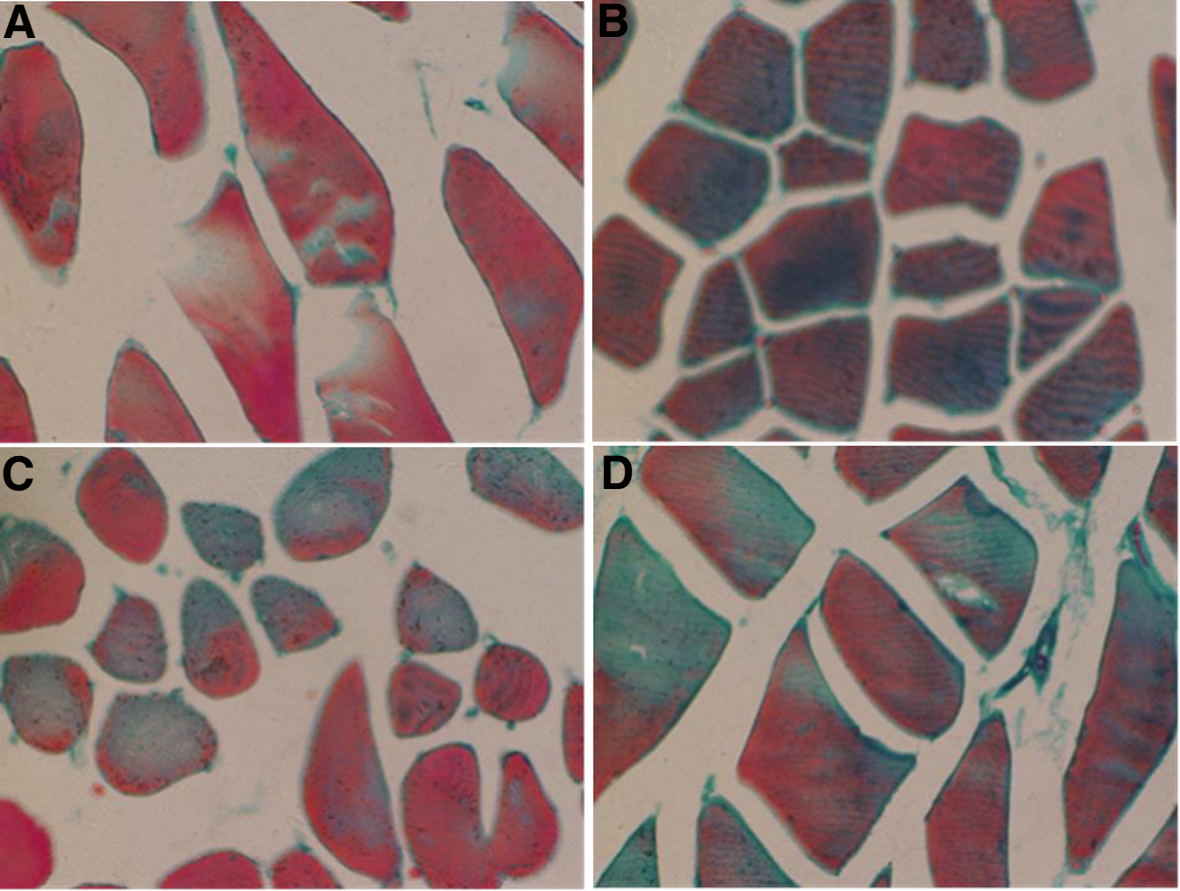
**Morphological changes of the skeletal muscle in control rats and COPD model rats that were treated with vehicles, AICAR or resveratrol at week 20 (H & E stained). (A)** Control rats; **(B)** COPD rats with vehicle; **(C)** COPD rats with AICAR treatment; and **(D)** COPD rats with resveratrol treatment. Amplification ×200.

**Figure 4:**
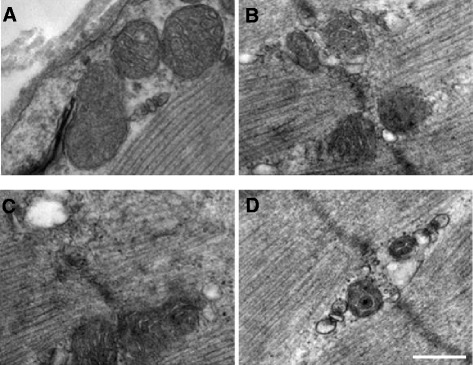
**Pathological changes of the mitochondria in the control rats and COPD model rats that were treated with vehicles, AICAR or resveratrol at week 20 (H & E stained). (A)** Control rats; **(B)** COPD rats with vehicle; **(C)** COPD rats with AICAR treatment; and **(D)** COPD rats with resveratrol treatment. Amplification ×100 000.

Interestingly, we found that the skeletal muscle fibers became atrophy in the COPD model rats, in comparison to those in the control rats. We also observed that there were more collagen depositions in the COPD rats (Figure 3B). Importantly, we found that both resveratrol and AICAR decreased the depositions of collagen in the skeletal muscle tissue in the COPD model rats (Figure 3C and D). Furthermore, the number of mitochondria in triceps tissue of COPD model rats was reduced and the mitochondria became swelling complicated with more vacuoles and the dissolved endometrial and crest of the mitochondria (Figure 4B). More importantly, both resveratrol and AICAR ameliorated the abnormal changes of mitochondria as mentioned above in the COPD model rats (Figure 4C and D).

### The increased levels of TNF-α in serum and skeletal muscle in COPD rats

**Figure 5:**
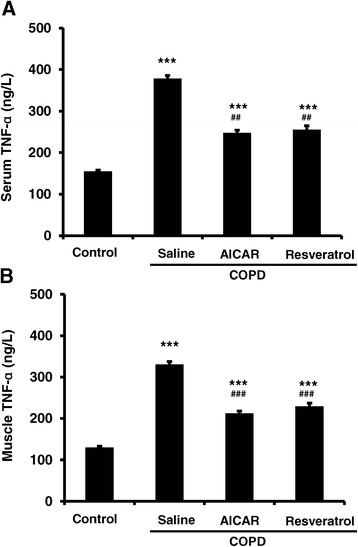
**The levels of TNF-α in both serum and muscle tissue were elevated in COPD model rats, and AICAR or resveratrol treatment decreased the COPD-based TNF-α elevation.** TNF-α in serum (**A**; Control: n = 14, 154.70 ± 3.47; COPD/saline: n = 12, 378.09 ± 7.60; COPD/AICAR, n = 14, 248.00 ± 6.30; COPD/Resveratrol, n = 12, 255.43 ± 9.40; ***F_3, 51_ = 181.12, *p* < 0.001 comparing to the control group; ###*F*_2, 37_ = 87.64, *p* < 0.001 for COPD/AICAR group or COPD/resveratrol group compared to the COPD/saline group). TNF-α in skeletal muscle (**B**; Control: n = 14, 129.77 ± 3.15; COPD/saline: n = 12, 330.56 ± 6.87; COPD / AICAR, n = 14, 212.37 ± 5.49; COPD/Resveratrol, n = 12, 229.30 ± 7.64; ***F_3, 51_ = 197.94, *p* < 0.001; *###F*_2, 37_ = 91.87, *p* < 0.001 for COPD/AICAR group or COPD/resveratrol group compared to the COPD/saline group).

Next, we tested if the TNF-α levels were also affected in the COPD model rats. We found that the levels of TNF-α in both serum and skeletal tissue were significantly increased in the COPD model rats in comparison to those in the control rats (Figure 5A). Interestingly, the levels of TNF-α in either serum or skeletal muscle tissue in the COPD model rats that were treated with resveratrol and AICAR were lower than those in COPD rats that received saline treatment (Figure 5), although we also noticed that the levels of TNF-α in the AICAR-treated rats were lower than those from the rats that were treated with resveratrol (Figure 5), yet the mechanisms need to be further studied.

### The decreased expressions of AMPK in skeletal muscle tissues in COPD rats

**Figure 6:**
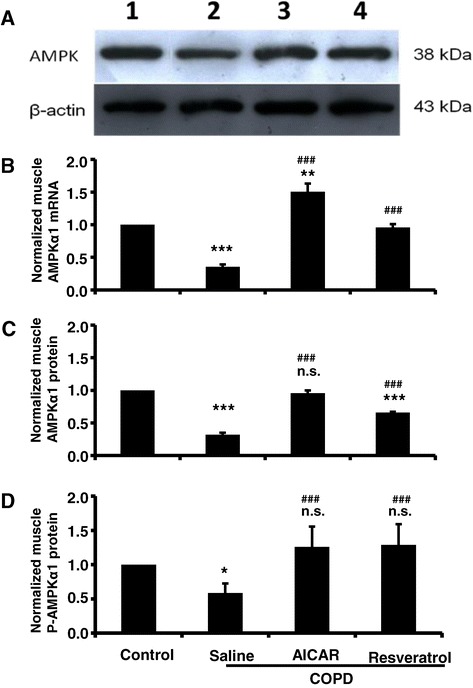
**The expression of AMPK in muscle tissue was decreased in COPD model rats, and AICAR or resveratrol treatment recovered AMPK expression in COPD model rats. (A)** Sample blots of AMPK and β-actin. Normalized muscle AMPK mRNA (**B**; Control: n = 14; COPD/saline: n = 12, 0.35 ± 0.04; COPD/AICAR, n = 14, 1.50 ± 0.12; COPD/Resveratrol, n = 12, 0.96 ± 0.05; ***F_1, 25_ = 68.31, *p* < 0.001 comparing the control group to the COPD/saline group; **F_1, 27_ = 11.89, *p* = 0.002 comparing the control group to the COPD/AICAR group; ###*F*_1, 23_ = 74.98, *p* < 0.001 comparing COPD/saline to COPD/resveratrol; ###*F*_1, 25_ = 60.98, *p* < 0.001 comparing COPD/saline to COPD/AICAR group). Normalized muscle AMPK protein (**C**; Control: n = 14; COPD/saline: n = 12, 0.32 ± 0.03; COPD/AICAR, n = 14, 0.96 ± 0.04; COPD/resveratrol, n = 12, 0.66 ± 0.01; ***F_1, 25_ = 66.17, *p* < 0.001 comparing the control group to the COPD/saline group; ***F_1, 25_ = 25.21, *p* < 0.001 comparing the control group to the COPD/resveratrol group; n.s. *F*_1, 27_ = 0.16, *p* = 0.70 comparing the control group to the COPD/AICAR; ###*F*_1, 25_ = 31.52, *p* < 0.001 comparing COPD/saline to COPD/AICAR; ###*F*_1, 23_ = 24.58, *p* < 0.001 comparing COPD/saline to COPD/resveratrol group. n.s. (not significant). Normalized muscle p-AMPK protein (**D**; Control: n = 6; COPD/saline: n = 6, 0.58 ± 0.14; COPD/AICAR, n = 6, 1.26 ± 0.30; COPD/resveratrol, n = 6, 1.29 ± 0.30; * *F*_1, 11_ = 5.1, *p* < 0.05 comparing the control group to the COPD/saline group; ###*F*_2, 17_ = 15.15, *p* < 0.001 comparing COPD/saline to COPD/AICAR and COPD/resveratrol. n.s. *F*_2, 17_ = 0.093 comparing the control group to the COPD/resveratrol group and COPD/resveratrol. n.s. (not significant).

Next, we examined if the expression levels of AMPK and p-AMPK were also affected in the COPD model rats, in according to that AMPK plays crucial functional roles in regulating energy metabolism [15]. We found that the expression levels of both AMPKα_1_ mRNA and protein in the skeletal muscle were significantly decreased in the COPD model rats, compared to those from the control rats and the COPD rats that were treated with AICAR and/or resveratrol (Figure 6A,B and C). As expected, the expression levels of the active p-AMPK were also significantly decreased in the COPD model rats and recovered with the treatment of AICAR and/or resveratrol (Figure 6D). We also noticed that the content of AMPKα protein in the resveratrol group was a little lower than that in the AICAR group (P <0.05), and further studies are needed to investigate the difference between them.

### The decreased expression of SIRT1 in skeletal muscle tissue in COPD rats

**Figure 7:**
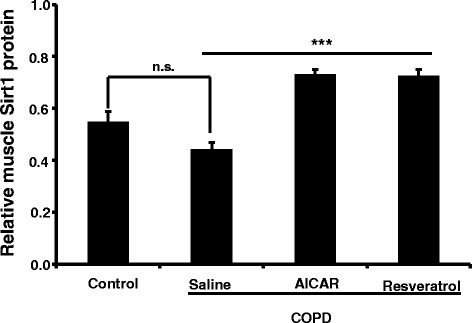
**AICAR and Resveratrol increased the expression of muscle SirT1 in muscle tissue in COPD model rats.** Muscle SirT1 (Control: n = 6; 0.55 ± 0.04; COPD/saline: n = 6, 0.44 ± 0.03; COPD/AICAR, n = 6, 0.73 ± 0.02; COPD/Resveratrol, n = 6, 0.73 ± 0.03). n.s. *F*_1, 11_ = 5.0, *p* = 0.051 comparing the control group to COPD/saline group. ***F_2, 17_ = 48.69, *p* < 0.001 comparing the COPD/saline group to COPD/AICAR and COPD/resveratrol group respectively. n.s. (not significant).

We next tested if the expression of SirT1 is also affected in the COPD model rats. We found that the expression levels of SIRT1 were slightly but not significantly decreased in COPD model rats. Meanwhile, we found that the expression levels of SirT1 protein in the skeletal muscle were significantly increased with the treatment of AICAR and resveratrol (Figure 7).

## Discussion

COPD is complicated with chronic inflammation that exerts crucial functional roles in the development and pathophysiology of COPD. Meanwhile, it has been recognized that the inflammation accompanying COPD leads to the skeletal muscle dysfunction. Strikingly, the skeletal muscle weakness has been recognized as one of the predictors for mortality in moderate or severe COPD patients [5]. The COPD-based dysfunctions of the skeletal muscle show the loss of body weight, decreased body mass index, muscle weakness and the decreased endurance, as well as the decreased resistance to fatigue. Previous studies demonstrated that systemic inflammation could exert influential effects on the energy metabolism of skeletal muscle [17], however it remains unknown that the underlying mechanism(s) of the abnormal energy metabolism in COPD patients. Consistent with the COPD patients, we found that the COPD rats also behaved restless and cough, as well as shortness in breathing at the early stage of COPD within 8 weeks (data not shown). With the development of COPD, the animals showed progressive and gradual reduction in the body weight (Figure 1) and more deposition of collagen fibers in the skeletal muscle in COPD rats (Figure 4). The morphology of mitochondria was abnormal as well, as indicated by the swelling, vacuolization, endometrial and damaged crest in the skeletal muscle mitochondria (Figure 4). These results indicated that the atrophy of skeletal muscle cells and fibrosis did occur in the COPD model rats, suggesting that the morphological abnormalities of mitochondria underlie the dysfunctions of the skeletal muscle energy metabolism partially explained why the COPD patients behave fatigue and decline in the exercise tolerance.

It has been well known that the TNF-α is a pro-inflammatory factor, because some of the inflammatory processes are mediated by the elevated levels of TNF-α. For example, previous studies indicated that the inflammation can be alleviated by inhibiting the activities of TNF-α [18]. We found that the expression levels of TNF-α in the peripheral blood were elevated in the COPD rats, in comparison to that from the control rats. Conversely, we also found that the expression levels of TNF-α were significantly decreased in the COPD rats that were treated with resveratrol or AICAR (Figure 5). Moreover, the expression levels of TNF-α in skeletal muscle were also significantly elevated in the COPD rats (Figure 5). Collectively, these results indicate that the lung inflammation complicated with the development of COPD might lead to the inflammation in the peripheral skeletal muscle tissue, which leads to the dysfunctions of the skeletal muscle. Meanwhile, it is reported that the morphological and functional abnormalities in mitochondria are mediated by the inflammation through a variety of signaling pathways [19,20]. Of note, it is also reported that AMPK regulates the functions of mitochondria, yet the underlying signaling pathway remains unknown [21]. Interestingly, our data indicated that the expression levels of AMPKα_1_ mRNA and protein were significantly reduced in the COPD model rats, in comparison to that from the control rats (Figure 6A,B and C). Meanwhile, the expression levels of the active p-AMPK were also significantly decreased in the COPD muscle (Figure 6D). We also found that the expression levels of muscular TNF-α and AMPK α_1_ were negatively correlated. We did not see any significant changes in the expression levels of SirT1 in COPD rats (Figure 7), which may also be involved in muscular energy metabolism [22]. AMPK is an important protein kinase in eukaryotic cells and exerts crucial functions in energy metabolism by the related metabolic enzymes [23,24]. Collectively, these results thus suggest that the dysfunction of skeletal muscular energy metabolism associated with the systemic inflammation in COPD rats was probably caused by decreasing the expression levels of AMPK although we cannot exclude other signaling pathways.

Moreover, we found that treatment of the COPD model rats with resveratrol or AICAR alleviated the morphological damage of mitochondria, in comparison to those in COPD rats with saline treatments (Figure 4). Resveratrol is a polyphenolic compound in grapes, red wine, purple grape juice, peanuts, and some berries. Previous studies indicated that resveratrol can reduce the release of proinflammatory cytokines and improve the levels of glutathione as well as reduce the MPO activity in lung tissue to thereby reduce lung injury [25]. The expression levels of TNF-α in peripheral blood and skeletal muscle are decreased in the COPD model rats with resveratrol or AICAR treatment, suggesting that resveratrol or AICAR might inhibit the inflammatory response in COPD rats. In both cases, we found that the expression levels of AMPK were significantly elevated in comparison to those in the COPD model rats that received saline, suggesting that resveratrol or AICAR may thus indirectly enhance AMPK activities through inhibiting the inflammatory response in addition to their direct activation on AMPK. Meanwhile, we also found that AICAR or resveratrol increased the expression levels of SirT1 in the COPD model rats (Figure 7). Previous reports indicate that AMPK and SirT1 interactively exert their roles in inflammation and metabolic regulation [26]. Also, there is evidence indicating that AMPK and SirT1 regulate each other and share some signaling pathways [27]. For instance, down-regulation of p-AMPK reduces the levels of SirT1 [28]; on the other way, SirT1 is required for moderate concentration of resveratrol to stimulate AMPK [29]. Based on our data, we propose that the changes of AMPK activities contribute to the dysfunctions of muscle metabolism in COPD rats, although we cannot exclude the possibility that SirT1 and other signaling are implicated in the modulation of muscular metabolism.

In summary, in according to the previous studies and our present findings, we proposed that in COPD model rats TNF-α may probably inhibit the AMPK expression in skeletal muscle. Meanwhile, resveratrol, as a new agent to reduce the release of proinflammatory cytokines, play a protective role in COPD. Therefore, AMPK may serve as a potential therapeutic target for the treatment of muscle weakness in COPD model rats.
